# Use of an Artificial Intelligence Algorithm to Increase Productivity in Implantable Loop Recorder Monitoring: A Multicentre Observational Study

**DOI:** 10.7759/cureus.107519

**Published:** 2026-04-22

**Authors:** Cherry Alexander, Alan Robertson, Sophie Bagnall, Catherine Vaughan, Andrew Saunders, Ken Teh, Faheem A Ahmad

**Affiliations:** 1 Department of Cardiology, Queen Elizabeth University Hospital Glasgow, Glasgow, GBR; 2 Department of Cardiology, Ninewells Hospital, Glasgow, GBR; 3 Department of Cardiology, West of Scotland Innovation Hub, Glasgow, GBR

**Keywords:** atrial fibrillation management, deep learning artificial intelligence, disruptive innovation, implantable loop recorder (ilr), unexplained syncope

## Abstract

Background: Insertable loop recorder (ILR) services are increasingly constrained by high volumes of transmitted episodes, many of which are false, clinically irrelevant, or non-actionable. Cloud-based artificial intelligence (AI) algorithms have the potential to suppress false atrial fibrillation (AF) and pause alerts while preserving clinically relevant events. We present the first real-world impact of an AI algorithm activated across a fixed multicentre ILR cohort.

Methods: We performed a retrospective, multicentre before-and-after cohort analysis of consecutive patients. Eligible patients had continuous ILR monitoring for 12 months before and 12 months after service-wide activation ("switch-on") of the Medtronic AccuRhythm AI platform (Medtronic plc, Galway, Ireland), enabling within-patient comparison. All clinician-facing transmitted alerts were counted in each period. Secondary analyses examined the concentration of alert burden across patients and estimated workflow impact using published time-and-motion data for remote transmission review. Differences in alert counts were tested using paired t-tests, reporting mean paired differences with 95% confidence intervals (CI).

Results: The cohort included 445 patients (Reveal LINQ n=438; LINQ II n=7); 440 (99%) were implanted for syncope (mean age 67±15 years; 49.6% male). Total transmitted alert volume fell from 4,261 pre-AI to 2,509 post-AI (29% reduction). The mean paired within-patient change was −3.94 alerts (95% CI −7.5 to −0.4; p<0.05). Alerts were highly concentrated: 7% of patients generated >90% of alerts, and 104 patients had intermittent disconnection from remote monitoring. Applying established workflow timings (11-13 minutes per remote transmission review) translated the reduction into 185-218 hours of physiologist review time released annually: approximately four hours per week of "virtual physiologist" capacity.

Conclusions: In routine practice, AI activation in a multicentre observational study was associated with a statistically significant reduction in ILR alert burden and a clinically meaningful release of staff capacity. Parallel management of high-alert patients and connectivity optimisation may further amplify the operational benefit.

## Introduction

Insertable cardiac monitors (ICMs) are a cornerstone technology for long-term rhythm surveillance in patients with suspected cardiac syncope and cryptogenic stroke [1,2]. ICMs, also known as implantable loop recorders (ILRs), are small devices around one-third the size of an AAA battery, implanted in the subcutaneous tissue [3]. Most contemporary models offer continuous monitoring of up to three years, with the capability for remote transmission. However, widespread uptake of ICM in clinical practice remains limited by operational challenges [4]. Conventional ICMs generate large volumes of alerts, many of which are false alerts, clinically irrelevant, or non-actionable recordings [5,6]. This data deluge strains already pressurised workflows, especially where clinic resources are already limited.

Machine learning algorithms offer the promise to streamline inappropriate alert generation, functioning in effect as a "virtual physiologist" (by enabling re-deployable physiology capacity) through filtering false positive alerts to reduce overall burden which enters the workflow. However, to date, no clear real-world evidence has been generated in a consistent patient cohort.

The AccuRhythm artificial intelligence (AI) platform (Medtronic plc, Galway, Ireland) is a cloud-based multi-layered convolutional neural network algorithm [7]. Transmitted atrial fibrillation (AF) and pause episodes are classified as either false or true/indeterminate. Episodes classified as false are suppressed and therefore "filtered out" from clinician view, while the remainder are available for clinician interpretation. The platform incorporates bypass logic intended to preserve potentially clinically important events (such as symptom correlated events, AF episodes of substantive duration (> 1 hour), infrequent pauses, and certain pause programming settings). In previous series, performance sensitivity is maintained while screening a high proportion of false positive alerts [8,9]. The algorithms have been trained utilising a database of one million electrocardiograms with supervised learning adjudicated by expert reviewers [10]. In May, 2025, the AI framework was switched on for all ILR patients, providing a unique opportunity to observe real-world implementation effects in a consistent patient cohort across NHS Scotland. 

The objective of this study was to evaluate the real-world impact of service-wide activation of a cloud-based AI alert-adjudication system on a fixed population of patients with an ILR across multiple NHS centres. The primary aim was to assess the change in clinician-facing alert volume before and after activation. Secondary aims were to examine how alert burden was distributed across the monitored cohort and the categories of alerts filtered and to estimate the potential workflow implications of any observed reduction in alerts using published time-and-motion data.

## Materials and methods

We designed a multicentre before-and-after observational study to quantify the impact of the "switch-on" of a machine-trained algorithm on alert burden in the ILR service. Four sites from NHS Scotland were included in the analysis (Queen Elizabeth University Hospital (Glasgow, UK), Glasgow Royal Infirmary (Glasgow, UK), Inverclyde Hospital (Glasgow, UK), Ninewells Hospital (Dundee, UK)). 

Consecutive ILR patients were eligible if they had been monitored for 12 months before and after AI activation on May 25, 2025, enabling within-patient comparison of transmitted episodes across the two periods (to ensure a meaningful post-activation period while retaining a consistent cohort). All ILR-transmitted episodes were extracted and counted in each period, with stratification by site. 

The primary outcome observed was alert volume per patient (and total cohort alerts) before and after AI activation. Secondary descriptive outcomes included the following: concentration of alert generation across the cohort (to identify the concentration of alert volume in patient subsets), connectivity status (intermittent disconnection from remote monitoring), and capacity release of cardiac physiology time. To evaluate the impact of changes in alert burden on the service, we estimated physiologist time savings by applying published time-and-motion workflow timings for ICM remote transmission review (including differentiation of actionable versus non-actionable review time where relevant) to the observed change in alert volumes [11]. We referenced earlier single-centre time-and-activity work showing remote transmission processing times of a similar magnitude (11.5 minutes per remote alert transmission), supporting the plausibility of the workload conversion approach [11]. Using these published per-transmission review time estimates, we calculated the total review-time difference associated with the observed reduction in alerts and expressed this as cumulative hours saved over the study period and as an indicative weekly capacity release of cardiac physiology time.

Alert counts were analysed as paired, within-patient data, with each patient serving as their own control. As all patients contributed equal observation time (12 months before and 12 months after AI activation), alert counts rather than rates were compared between periods. Between-period differences in alert counts (overall and by site) were tested using a paired t-test, reporting the mean paired difference with 95% confidence intervals (CI) and corresponding p-values for the within-patient change. Statistical significance was defined as p<0.05. Site-level results were examined descriptively to assess potential heterogeneity in implementation effect, recognising that local programming practices, enrolment workflows, and connectivity variations may influence alert generation and the degree of AI-enabled false alert suppression. Operational factors relevant to real-world remote monitoring performance were also examined, including intermittent disconnection from remote monitoring (as indicated by gaps in monitoring connectivity status); this influence observed alert volumes and clinic workload independent of algorithm effects. Given the skewed distribution typical of count data, a Wilcoxon signed-rank test was performed as a sensitivity analysis to confirm the robustness of findings. We also conducted a one-way ANOVA to formally assess whether the magnitude of reduction differed between sites. Analyses were performed using R (Version 4.5.2, R Foundation for Statistical Computing, Vienna, Austria).

This analysis used routinely collected remote monitoring outputs focused on episode and alert counts and operational monitoring characteristics, without any interventional change to patient care beyond standard-of-care platform activation. The project was conducted in accordance with local governance requirements for retrospective service evaluations using routinely generated clinical monitoring data.

## Results

A total of 445 patients were included (Reveal LINQ n=438; LINQ II n=7). The predominant implantation indication was syncope (440/445; 99%). The mean age was 67 years (SD±15), and 218 (49.6%) were male. 

**Figure 1:**
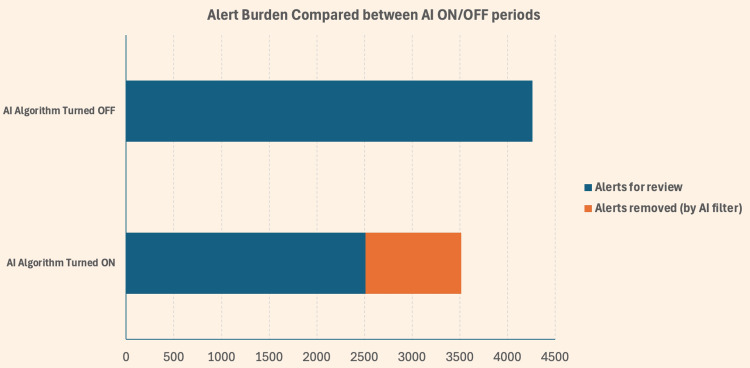
Aggregate reduction in alert burden before and after the "switch-on" of AI algorithm AI: artificial intelligence

Overall, the total number of clinician-facing alerts reviewed decreased from 4,261 prior to AI activation to 2,509 following AI activation. This equates to a 41.1% relative reduction in clinician-facing transmitted alerts reviewed, with a statistically significant overall intervention effect by paired testing (Figure 1). During the post-AI period, a further 1,007 alerts were suppressed by the AI algorithm which would have otherwise entered the clinical workflow. This corresponds to an AI suppression rate of 28.6% of potential alerts in the AI-on period (1,007 of 3,516 total potential alerts).

Paired within-patient analysis demonstrated a statistically significant reduction in alert burden after AI activation. The mean paired difference was −3.94 alerts per patient (95% CI −7.5 to −0.4; p=0.026; paired t-test). The magnitude of reduction varied between sites, suggesting heterogeneity in real-world implementation and warranting further evaluation of local workflow and programming factors (Table 1). Findings were consistent using non-parametric testing (Wilcoxon signed-rank p=0.018).

**Table 1:** Alert reduction across multiple health boards AI: artificial intelligence; AF: atrial fibrillation

Site	Patients	AI algorithm turned off	AI algorithm turned on								
	N	Viewed alerts	Viewed alerts	Removed	Reduction	Mean paired reduction (95% CI)	P-value	Alerts removed (by category)		Projected time savings	
								False AF alert	False pause	Hours	Hours/week
1	93	2,360	1,203	599	33%	12.40 (−2.29 to 27.20)	0.97	498	101	113	2.2
2	242	1,716	1,100	339	24%	2.55 (−0.66 to 5.75)	0.119	323	16	64	1.2
3	56	112	147	31	17%	−0.62 (−2.41 to 1.16)	0.487	30	1	6	0.1
4	54	73	59	38	39%	0.26 (-0.38 to 0.90)	0.442	36	2	7	0.1
Total	445	4,261	2,509	1,007	29%	3.94 (0.41 to 7.46)	<0.05	887	120	190	3.6

Alert generation was highly concentrated within the cohort: 7% of patients accounted for more than 90% of all transmitted alerts, highlighting a potential "high-transmitter" subgroup (generating more than 10% of alerts). In addition, 104 patients experienced intermittent disconnection from remote monitoring during the study period. Applying published time-and-motion estimates for remote transmission review (11-13 minutes per alert), suppression of 1,007 alerts by the AI algorithm was estimated to release approximately 185-218 hours of physiologist review time annually, equivalent to 3.6-4.2 hours per week of re-deployable service capacity. 

## Discussion

In this multicentre observational cohort study, service-wide activation of an AI algorithm was associated with a statistically significant suppression of clinician-facing ILR alert burden, leading to a substantial release of additional staff capacity. This magnitude is operationally meaningful in services where ILR remote monitoring generates high transmission volumes and competes with other diagnostic and therapeutic device follow-up activities.

ILR services are workload-intensive compared with therapeutic device follow-up, largely driven by high transmission volumes. Multicentre time-and-motion analyses demonstrate that remote transmission review typically consumes 11-13 minutes per alert transmission, while in-person consults require substantially more time [11]. The cumulative demand of ILR monitoring on cardiac physiology staff time can easily reach several hours per patient-year due to frequent transmissions. Clinically relevant transmissions may require longer review than non-actionable results, while poor connectivity and non-compliance further increase administrative burden. In this context, even modest absolute reductions in alert volume translate into meaningful operational service capacity, particularly when suppression targets high-volume alert categories such as false AF and pause alerts.

The overall reduction observed in our observed series is directionally consistent with reductions reported in controlled validation settings. Seiler and colleagues demonstrated a reduction of 35% using a proprietary ICM algorithm in a limited series [11]. Although our primary signal is shown at the aggregate multicentre level, site-level impact remains operationally relevant, since alert review burden is often experienced within local physiology workflows. In this study, patient numbers at each individual site were modest, limiting power to detect statistically significant within-site differences even where the direction of effect was favourable. Site-level differences may reflect ongoing local heterogeneity in programming, workflow, and connectivity which were not captured. The pooled cohort still remains a robust estimate of the overall service-level association between AI activation and reduced alert burden. The current algorithm incorporates safety-oriented bypass rules (e.g. symptom-correlated events and prolonged AF episodes) that intentionally preserve clinically significant events for clinician review, thereby reducing the proportion of alerts eligible for suppression. In addition, local factors (such as programming choices and distribution of alert types) will influence the measured effect size, making a lower net reduction at the service level plausible even when algorithmic performance is stable.

Alert generation was highly concentrated in a small cohort of patients, with 7% of patients accounting for >90% of alerts. In addition, 104 patients were intermittently disconnected from remote monitoring. These findings identify two additional operational targets for further improvement beyond AI-based alert adjudication alone [12]. Connectivity failures and incomplete transmissions from "high-transmitter" patients generate additional workload not fully captured by simple "minutes per transmission" timing metrics. Targeted review of this subgroup, including assessment of signal quality, programming parameters, symptom activation practices, and underlying clinical rhythm phenotype (such as frequent ectopy or sinus arrhythmia), could help further reduce alert burden. Proactive connectivity solutions may also improve service administration efficiency, although prospective validation of specific strategies has yet to be demonstrated in multicentre series. A combined strategy integrating AI filtration with targeted operational interventions will therefore likely amplify AI-related efficiencies and improve the continuity of diagnostic surveillance, leading to maximised efficiency gains.

These results further highlight that the total cost of ownership in ILR services is not driven by device unit price alone. AI-enabled alert suppression can release meaningful physiologist capacity by filtering out non-actionable reviews. As these productivity gains are embedded in software capability and "soft" intellectual property of vendors rather than hardware components, they may not be reflected in traditional procurement comparisons focused solely on unit price. Procurement frameworks should therefore incorporate these operational outcomes to avoid selecting solutions which appear cheaper upfront but impose higher recurrent staffing costs and constrain service growth.

Workflow efficiency remains critical to the sustainability of ILR programmes. Prolonged rhythm monitoring increases the detection of actionable arrhythmias in cryptogenic stroke and syncope populations [13], but scalable ILR services require manageable alert volumes. As healthcare systems seek to expand secondary stroke prevention and syncope detection strategies, AI-enabled ICM technology offers the ability to deploy such technology in a workflow-efficient fashion to maintain operational feasibility in a resource-constrained system. 

Limitations

This work has several limitations. First, it employed an observational before-and-after design rather than a randomised study with a control group. Confounders relating to patient population differences will still be potentially present, despite the workflow being conceptually reproducible. Second, site-to-site variation in implementation such as implant technique, local programming practices, and patient education (including symptom-trigger usage policy) all likely contributed to heterogeneity in observed effect size. Third, workflow efficiency was estimated using published time-and-motion data rather than prospectively measured within the participating centres. Finally, while alert suppression was quantified, downstream clinical outcomes were not assessed. Future work should pair algorithm activation with prospective workflow measurement and the evaluation of clinical impact. Further evaluation using a contemporaneous control group would help improve attribution of observed reductions solely to the activated AI platform. The observed effect was also specific to this vendor-specific AI system, the specifics of which remain proprietary. Therefore, while these results should not be assumed to apply uniformly across other vendors or algorithms, they help frame the wider discussion around the potential role of AI in improving productivity and releasing capacity within remote monitoring services. 

Despite these limitations, the findings support AI-assisted suppression of false alerts as a potentially practical capacity intervention, functionally enabling a "virtual physiologist" (in a very limited sense), capable of maintaining clinical oversight while improving service efficiency in resource-constrained ILR services. 

## Conclusions

In this multicentre evaluation, activation of AI-based alert adjudication was associated with a significant reduction in clinician-facing ILR alerts and a measurable release of physiologist review capacity. This translation of re-deployable service capacity can be relocated to higher-value patient care. A prospective controlled evaluation is needed to definitively confirm the extent to which this effect is directly attributable to the intervention. The value of modern ICM platforms extends beyond hardware to AI-enabled software and workflow efficiencies; procurement decisions should explicitly account for these productivity gains to avoid underestimating both total costs and service-efficiency benefits.
